# Safety and Efficacy of Two Different Concentrations of Ketamine and Propofol Combinations in Cataract Surgery: A Double-blind Randomized Clinical Trial: Efficacy of Two Different Concentrations of Ketofol in Cataract Surgery

**DOI:** 10.31661/gmj.v11i.2744

**Published:** 2022-12-26

**Authors:** Hamidreza Shetabi, Seyed Morteza Heydari, Amir Shafa, Alireza Peyman, Maryam Najafiani

**Affiliations:** ^1^ Department of Anesthesiology, Anesthesiology and Critical Care Research Center, Isfahan University of Medical Sciences, Isfahan, Iran; ^2^ Department of Ophthalmology, Ophthalmology Research Center, Isfahan University of Medical Sciences, Isfahan, Iran; ^3^ Student Research Committee, Medical School, Isfahan University of Medical Sciences, Isfahan, Iran

**Keywords:** Sedation, Ketamine, Propofol, Ketofol, Cataract

## Abstract

**Background:**

Due to the importance of appropriate sedation and immobility of the patient in cataract surgery, this study was performed to compare the safety and efficacy of a combination of ketamine and propofol (ketofol) in two different ratios.

**Materials and Methods:**

This double-blind, randomized clinical trial was carried out on patients who underwent cataract surgery in Feyz Hospital, Isfahan, Iran. Patients were randomly divided into group I (n=30, ketamine/propofol 2:1 ratio) and group II (n=30, ketamine/propofol 4:1 ratio). The quality of sedation (using Ramsay sedation scale [RSS]), cardiovascular parameters such as systolic blood pressure (SBP), diastolic blood pressure (DBP), arterial blood pressure, SPO2, and heart rate, as well as patient and surgeon satisfaction, were evaluated in both groups.

**Results:**

The SPO2 and heart rate were significantly lower and higher in group I than in group II during various surgery times, respectively (P=0.0001 for both comparisons). In terms of patient and surgeon satisfaction, it was found that no patient was dissatisfied with the sedation status in group II, while four patients (13.3%) in group I were dissatisfied (P=0.005). However, RSS, SBP, and DBP were significantly different between the two groups (P0.05 for all comparisons).

**Conclusion:**

It seems that the use of lower ketamine doses in combination with propofol (4:1) is a safe and preferable option to provide sedation in cataract surgery.

## Introduction

Cataract is a turbidity in the eye lens that causes vision loss in more than 80 million individuals worldwide [1]. It is also an important factor in blindness and visual loss, and cataract surgery is one of the most common procedures performed globally [2].

Because pain during local anesthesia can lead to complications, analgesics or painkillers must be used to alleviate it. Opioids, propofol, and benzodiazepine medications have been selectively utilized to reduce patients' anxiety and pain [3][4]. New compounds, such as the ketamine-propofol combination (ketofol) may be able to replace the previous medicinal regimen [5]. Propofol is a non-opioid and non-barbiturate anesthetic drug with anti-nausea properties, with side effects such as dose-dependent respiratory and cardiovascular function suppression. Propofol has a short onset of action, followed by a short duration to improve the patient's recovery, which is about 10 to 20 min [6].

Ketamine is a phencyclidine (PCP) derivative known as an effective anesthetic agent, which induces sufficient analgesia and amnesia. The combination of ketamine and propofol has been used successfully. Several studies have reported that the combined effects of these two medications are effective and safe for sedation [7][8]. However, the effects of their various combination ratios on patients with cataract surgery have yet to be completely examined. This study aimed to investigate the sedation quality of two different ketofol ratios (2:1 vs. 4:1), as well as hemodynamic responses, side effects, and patient and physician satisfaction to establish the lowest safe and effective ketofol dosage.

## Materials and Methods

### Patients

This double-blind, randomized clinical trial was performed on 60 patients with cataracts who were referred to the Faiz Hospital affiliated with Isfahan University of Medical Sciences from March 2020 to March 2021.

### Ethical Considerations

This study was approved by the Ethics Committee of the University (approval code: IR.MUI.REC.1396.3.628) and registered at the Iranian Clinical Trial Center (registering code: IRCT20180416039326N4). Also, informed consent was obtained from all the patients

### Inclusion and Exclusion Criteria

Patients over 18 years old with informed consent and grouped in terms of physical status in Class I or II of the American Society of Anesthesiologists were included in the study [9]. The exclusion criteria were a history of allergy to ketamine and propofol, egg, or soya, as well as alcohol, opiate, or benzodiazepine abuse, pregnancy, glaucoma, evidence of increased intracranial pressure, psychosis, schizophrenia, active upper respiratory tract infection or asthma, and chronic lung disease. The patients with any complications that could affect the anesthesia program were excluded.

### Randomization and Blinding

On the surgery day, a nurse who was not a research team member divided the patients into two parallel groups (n=30 per group), each receiving two different ketofol ratios. Patients, surgeons, and data collectors were blinded regarding the sedation regimen in the two groups. Using a computer-based algorithm that followed a random number generator technique, patients were randomly divided into two groups with a 1:1 aspect ratio to receive either Ketofol (2:1 ratio) or Ketofol (4:1 ratio). The participants were categorized using an online calculator at www.calculator.net, and each patient was randomly allocated a number depending on the calculator's output. Numbers 1 to 30 were in the Ketofol (2:1 ratio) group, whereas numbers 31 to 60 were in the Ketofol (2:1 ratio) group.

### Groups and Interventions

Prior to surgery, the patients fasted for eight hours. An anesthesiologist anesthetized all the patients, and a surgeon conducted the surgeries. An anesthesiologist prepared the following drug regimens with no role in the data-gathering process.

Group I (2:1): A mixture was prepared using 200 mg of propofol (10 mL) combined with 100 mg of ketamine (2 mL).

Group II (4:1): A mixture was prepared by adding 10 mL of propofol 2% (200 mg) with 1 mL of ketamine (50 mg/mL).

At the time of surgery, ketofol was infused with a syringe pump (B/Braun), an initial bolus dose of 0.6 mg/kg, and an infusion rate of 50-100 µg/kg/min (calculation adjusted based on propofol dose) to achieve a Ramsay sedation score (RSS) [10] of three.

### Outcomes

Duration of anesthesia, surgery, recovery, satisfaction with the surgeon at the end of the surgery, and patient satisfaction with the sedation quality were all evaluated based on the Likert criterion and then recorded before the patient was transferred to the ward.

### Statistical Analysis

The data were finally imported into SPSS software version 26.0 for Windows (IBM Corp., Armonk, N.Y., USA). In this analysis, the Shapiro-Wilk test was used to check the normality of data. The alpha error of 5% (95% confidence interval [CI]) was taken as the limit of rejecting or confirming the null hypothesis, and all mean comparison tests were performed as two-tailed tests. All continuous and categorical variables were expressed as mean±standard deviation and numbers (percentages), respectively. Moreover, Mauchly's sphericity test was used to check variance. Data were analyzed using chi-square, Mann-Whitney U, and One-Way Repeated Measures ANOVA, followed by the Bonferroni test for multiple comparisons. The significance level was considered at P=0.05.

## Results

**Figure-1:**
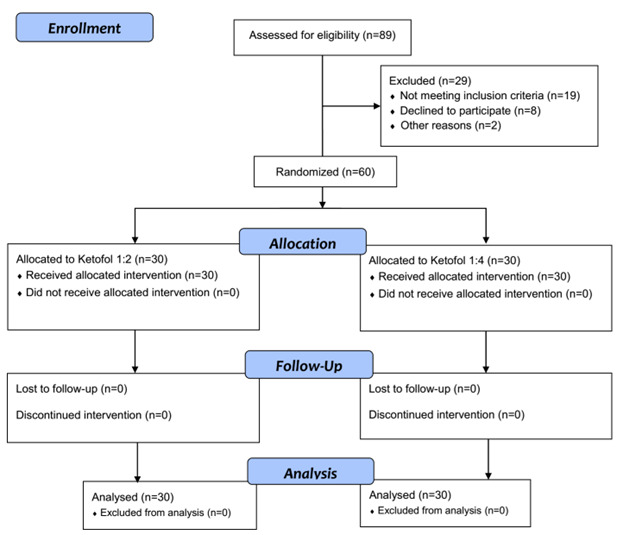
Figure 1. Flowchart of the participants.

**Table:** Table1. Baseline Characteristics of Studied Patients

**Variables**	**Group I**	**Group II**	**P-value**
**Sex, n(%)**			
Male	13 (43.3)	17 (56.7)	0.3
Female	17 (56.7)	13 (43.3)
**Age, y (mean±SD)**	66.30±13.47	62.03±11.6	0.54
**Weight, Kg (mean±SD)**	71.47±14.62	74.07±12.64	0.26
**Height, cm(mean±SD)**	161.2±18.59	165.23±6.66	0.38
**BMI, kg/m ^2^ (mean±SD) **	29.22±5.05	27.27±5.07	0.26
**ASA, n(%)**			
1	12 (40)	14 (46.6)	0.6
2	18 (60)	16 (53.4)

**BMI:** Body mass index; **ASA:** American society of anesthesiologists

**Table:** Table2. Hemodynamic Changes and Ramsay Sedation Scale Score Among Studied Patients

**Variables**	**Time points**	**Groups**		**P ^1^**	**P ^2^**	**P ^3^**	**P ^4^**
		**I**	**II**				
**SBP (mmHg)**	Before surgery	148.5±15.79	143.67±16.6	0.253			
	During surgery	148.98±15.12	140.85±21.05	0.029	0.348	0.034	0.569
	After surgery	147.58±14.1	138.07±17.06	0.022			
**DBP (mmHg)**	Before surgery	84.87±6.99	86.9±10.43	0.906			
	During surgery	85.87±7.62	86.78±12.38	0.706	0.143	0.744	0.37
	After surgery	84.78±8.24	84.03±11.4	0.756			
**Arterial blood pressure (mmHg)**	Before surgery	101.15 ±22.28	104.77±10.59	0.336			
	During surgery	104.98±23.02	104.88±15.86	0.203	0.181	0.759	0.507
	After surgery	101.42±24.1	102.02±12.93	0.141			
**SPO_2_ (%) **	Before surgery	95.55±3.32	96.65±1.28	0.04			
	During surgery	97.8±1.24	98.43±1.15	0.036	<0.0001	0.019	0.479
	After surgery	98.13±1.64	98.47±1.07	0.681			
**Heart rate (bit per min) **	Before surgery	77.6±15.59	73.27±15.3	0.282
	During surgery	76.55±12.41	70.68±13.4	0.084	<0.0001	0.223	0.167
	After surgery	71.93±11.64	69.96±12.4	0.529			
**RSS**	5^th^ minutes	2.83±0.44	2.98±0.44	0.14			
	10^th^ minutes	4.93±0.43	4.88±0.58	0.908	0.072	0.525	0.133
	15^th^ minutes	2.03±0.7	1.78±0.55	0.275			

Data presented as mean±SD **SBP:** Systolic blood pressure; **DBP:** Diastolic blood pressure; **RSS:** Ramsay sedation scale; **P^1^:** Significance level of the difference between the two groups in each time period according to the independent sample t-test/Mann-Whitney U. **P^2^:** Assessing time effect by using One-way ANOVA **P^3^:** Assessing group effect by using One-way ANOVA. **P^4^:** Interaction of time and group by using One-way ANOVA

In this study, 60 patients who underwent tranquilizing cataract surgery were divided into two groups (Figure-1) and received sedation by ketofol infusion in a 2:1 and 4:1 ratio, respectively. No patient was excluded due to an unwanted incidence of side effects. The two groups had no significant difference in terms of their basic and demographic variables such as age, sex, weight, height, body mass index (BMI), and American society of anesthesiologists (ASA) classification (Table-1).

Table-2 demonstrates the comparison between the two groups in terms of their hemodynamic changes at the study times as well as mean RSS during surgery. According to the ANOVA test, the mean of systolic blood pressure (SBP), diastolic blood pressure (DBP), and arterial blood pressure were not different between the two groups in terms of different times, groups, and interaction of time and groups (P>0.05 for all comparisons). Similarly, the RSS showed no significant difference between the two groups at any comparison (P>0.05 for all comparisons). However, the SPO _2_ and heart rate were significantly lower and higher in the ketofol 2:1 group than in the ketofol 4:1 group during different surgery times, respectively (P=<0.0001 for both comparisons). Accordingly, based on the Bonferroni test for multiple comparisons, SPO_2_ showed a significantly lower rate than the rates during and after surgery (P<0.0001 for both comparisons). Moreover, the mean heart rate after surgery was significantly lower than the mean values before and after surgery (P=0.001 and P=0.015, respectively). Notably, regarding the interaction between time and group, none of the parameters were significantly different between the two groups (P>0.05 for all comparisons).

In terms of patient and surgeon satisfaction, it was observed that in group II, no patient, while in group I, four patients (13.3%) were dissatisfied with the sedation status. In terms of patient sedation conditions, it was found that three physicians in group I were dissatisfied, while no dissatisfaction was recorded in group II. In quantitative analyses, patient satisfaction was significantly lower in group I (4.37±1.03) than in group II (P<0.001). However, there was no significant difference in surgeon satisfaction between group I and group II (4.17±1.23 vs. 4.57±0.62, P=0.301).

The assessment of the drug's side effects indicated that two patients from group I and one from group II experienced nausea and vomiting before surgery; however, the difference between the two groups was insignificant (P=0.55). During the operation, no patient suffered from hemodynamic disorders. The mean duration of surgery in groups I and II were 13.93±2.7 and 14.24±3.2 minutes (P=0.612), respectively. The mean duration of stay in the post-anesthesia care unit (recovery) in groups I and II were 35.3±11.5 and 32.2±8.8 minutes, respectively (P=0.32).

## Discussion

In the present study, we compared the effects of 2:1 and 4:1 propofol-ketamine combination ratios during cataract surgery. Our findings revealed that the satisfaction level of patients and surgeons in the group that received a 4:1 propofol-ketamine ratio was significantly higher than in the first group. However, there was no significant difference between the two groups in terms of RSS, cardiovascular responses, duration of surgery, surgeon satisfaction, and length of stay in recovery. No serious events were in any group, and thus the two ketofol combinations appear similarly safe.

Regarding patient and surgeon consent, the lower satisfaction level in group I might be related to the side effects of the high ketamine doses, such as restlessness, lack of cooperation during surgery despite deeper sedation, and post-operative nausea. In this respect, Daabiss *et al*. evaluated the analgesic quality and side effects of different concentrations of ketofol in children who underwent different surgeries such as esophagoscopy, rectoscopy, bone marrow aspiration, and liver biopsy [11]. They showed that patients who received propofol-ketamine in a ratio of 1:1 experienced more nausea and vomiting, psychological complications, and higher recovery time compared to the group receiving propofol-ketamine in a ratio of 4:1 [11], which confirms our findings.

Wang *et al*. investigated the effect of 2:1, 3:1, and 4:1 propofol and ketamine ratios, propofol-fentanyl, and propofol alone on the level of sedation, hemodynamic changes, and withdrawal time from the recovery [12]. The ketofol groups had high efficacy in abortion candidates. They concluded that ketofol was as effective and safe as the propofol-fentanyl combination, particularly in 3:1 and 4:1 ketofol ratios in patients following abortion [12]. Moreover, in Salem *et al*. [13] study, a low dose of ketofol was investigated in endoscopic applications in obese patients. They indicated that two concentrations of ketofol (2:1 and 4:1) were safe and effective for sedation and anesthesia in patients with obesity, and the 4:1 ketofol combination reduced the psychological side effects and the clearance time [13]. Likewise, the sedation quality and side effects of 2:1 and 3:1 ketofol ratios were studied in 60 children undergoing lumbar puncture and bone marrow aspiration procedures [14]. It was shown that the lower ketamine doses had fewer physiological side effects and less recovery time [14].

Coulter *et al*. [15] evaluated different ketofol ratios for general anesthesia in children and concluded that when the amount of ketofol infusion is not reduced, the duration of recovery increases. They suggested an optimal 1:5 ketamine to propofol ratio for 30 minutes of anesthesia and ratios of 1:6 and 1:7 for 90 minutes of anesthesia [15]. Also, they evaluated ketofol in different ratios for sedation in another group of children undergoing surgery. They suggested the 1:3 ketamine-propofol ratio as the best combination for alternative doses [15]. According to this study, the optimal ketofol dose for the children was initially 0.1 mL/kg, followed by 0.05 mL/kg for two minutes, and then 0.025 mL/kg for the subsequent doses [15]. In another study, the optimal ketofol dose for adults was 0.05 mL/kg and then 0.025 mL/kg for the subsequent doses [16]. In addition, a ratio of 3:1 leads to a prolonged recovery [16].

Another study assessed the effects of different propofol-ketamine ratios (1:1 vs. 1:3) on analgesia following nose fractures [17]. Their findings showed no differences in the hemodynamic parameters between the two groups. However, hallucinations, vomiting, and recovery time in the group that received a lower ketamine concentration were reduced [17].

The results of the present study were in line with those of the previous studies in terms of higher satisfaction and fewer post-operative complications after propofol-ketamine combination therapy with a low proportion of ketamine [13][14][15][16][17][18].

The evaluation of hemodynamic and vital parameters during the surgery and recovery revealed that neither of the above-mentioned compounds had any side effects on the patient's vital signs. The results also revealed no severe case of hemodynamic disorder requiring medical intervention and that the patient's exclusion from the study was observed; however, there was a significant difference between the two groups regarding the mean heart rate at a one-time point). The two groups showed a significant difference in the oxygen saturation level. The oxygen saturation in the propofol-ketamine group with a ratio of 2:1 was lower, but the exhaled carbon dioxide was not different between the two groups. In agreement with the findings of the present study, Aydogmus *et al*. [18] investigated the two 2:1 and 4:1 propofol-ketamine ratios in patients undergoing colonoscopy. They indicated that the 2:1 ratio provided more suitable hemodynamic conditions, but in general, no significant difference was observed between the two groups in terms of hemodynamic impairment [18].

Our study had some limitations as it was performed on a small group of patients undergoing cataract surgery. We did not include patients with an ASA value greater than two. Moreover, the quality of sedation was assessed objectively. Therefore, the results of the present study may not be generalizable to other surgical procedures, races, or countries.

## Conclusion

Patients and surgeons were more satisfied with ketofol in a 4:1 ratio compared to a 2:1 ratio with similar sedation during the cataract surgery without hemodynamic and respiratory suppressions. It seems that using a lower ketamine dose in combination with propofol (4:1) is a safe and effective approach as the preferred option to provide sedation in cataract surgery. However, further studies are recommended due to the limitations of our study, e.g., the limited sample size and the single center of the study site.

## Conflict of Interest

The authors declare that they have no conflict of interest.
